# Transition from Curative to Palliative Care in Cancer

**DOI:** 10.4103/0973-1075.78442

**Published:** 2011

**Authors:** Jaspreet Kaur, Bidhu K Mohanti

**Affiliations:** Department of Radiation Oncology, Dr. BRA Institute Rotary Cancer Hospital, All India Institute of Medical Sciences, Ansari Nagar, New Delhi, India. E-mail: drbkmohanti@rediffmail.com

The medical community, especially the doctors and nurses engaged in cancer care, recognize that a proportion of the patients either at presentation or during the course of therapy will show advanced and progressive disease.[1–4]

In real life, every individual learns how to move from childhood to adulthood, to be a parent and to enter old age. The transition or shift is considered integral to our living. Similarly, treatment of many diseases often requires a change from curative to a palliative approach and a shift in our goal of medical care. Yet, the transition between palliative and curative therapy may be filled with uncertainties for the patients, their families and health professionals. Healthcare systems face these concerns because, i) there are no clearcut boundaries between curative and palliative care, and ii) physicians and nurses often lack the knowledge of what palliative care actually involves.[1] Patients with advanced cancer face difficult decisions regarding their treatment and end-of-life care, because although options for cancer-directed therapies (CDT) are increasingly available, few provide long-term cure.[2] Medical professionals should be trained and well-skilled towards achieving a right and ethical balance between aggressive CDT and the medical care of patients when they reach the terminal stage of disease.[4]

## CHANGING THE GOAL IN CANCER CARE

Mr. KS, a 65-year-old retired metal worker, was diagnosed with locally advanced (Stage IIIB) non-small cell lung carcinoma of left side. He was treated with three cycles of platinum-based chemotherapy followed by local radiotherapy to the lung; over a treatment course of five months. His chemotherapy was stopped after three cycles due to poor tolerance and prolonged low blood counts. Three months after completion of his treatment and eight months after the cancer diagnosis, he has again shown rapid decline in general heath. Presently he has progressive breathing difficulty, loss of appetite, pain in chest and frequent sleep disturbances. The chest computed tomography (CT) scan reveals pleural effusion and persisting primary lung tumor. His wife and children are now keen to know his present disease condition and have a few pertinent questions – “should he take further second-line chemotherapy, and how much of relief can he get from chemotherapy or other forms of medical care?”

The above case scenario is a common clinical situation encountered by most physicians and nurses in cancer practice. Yet, it poses several complex questions for the physicians, patients, and the caregivers. Whether the clinicians will accept the disease as incurable and will forego further second-line anti-cancer therapy? If so, can these facts be communicated to the patient and his family effectively? If yes, will Mr. KS and his family accept this transition from cure to palliation?

Second-line treatments, in the form of chemotherapy, radiotherapy and surgery, for many cancers, have been explored with limited benefits. Neoplasms where second-line chemotherapy is commonly used are ovary, breast, colon, and lung. For example, three drugs are approved for patients who progress after platinum-based chemotherapy in advanced lung cancer: docetaxel, pemetrexed and erlotinib. These agents have similar efficacy in terms of response and survival, but have different toxicity profiles. Patients who have a good performance status benefit the most with a median survival of nine months, although eventually all patients experience disease progression.[5] The economic cost of lung cancer is high, and it is becoming increasingly important to deliver consistent and cost-effective care.[6] The explosion of new drugs has been accompanied by dramatic increases in cost, often without significant improvement in patient survival. Ongoing delivery of chemotherapy, first line followed by second line, may lead to adverse events and toxicities, and chemotherapy recipients incur large incremental expenditures for the treatment-related events, one recent evidence indicating to >$17000 per year.[7] It has been observed that the newer drugs are usually experimental and costly, often there are no standard second-line regimens, and result in a heterogeneous response for a progression-free survival ranging from a few weeks to six months.[8]

Radiotherapy, for symptom palliation and for restraining the tumor progression, is commonly employed in advanced, recurrent and metastatic cancers. The radiotherapy fractionation and course is kept to a minimum. Single-fraction treatment is often used to treat painful bony metastases. Hypofractionated radiotherapy (1-10 fractions) has been used in lung, head and neck, gastrointestinal, genitourinary cancers and in brain metastases with symptom response rates ranging from 40-97% and survival improvement between three to six months. Palliative radiotherapy is often more cost-effective and less toxic compared to systemic chemotherapy.[9] Re-irradiation is selectively utilized for disease failure, when patient has good performance status and the tumor is suitable for limited field radiotherapy again.[10]

Salvage surgery to increase the disease control and survival is undertaken, after failure of primary chemotherapy or radiotherapy, in cancers of head and neck, lung, gastrointestinal, genitourinary, Central Nervous System and bone and soft tissue origins.[11] However, the practice is mainly done in a limited manner at experienced institutions with careful patient selection.

Mr. KS has poor health and multiple symptoms due to rapid disease progression and further treatment like chemotherapy, radiotherapy or surgery are no longer feasible options for him. A discussion on patient’s goals and medical needs for providing him comfort from the above distressing symptoms are planned with Mr. KS and his family members.

Important issues in the transition from curative treatment to palliative care are agreement, timing and decision-making. When curative treatment is no longer an option, a decision to start palliative care should be made simultaneously.[3]

In this context it is relevant to refer to the study involving physicians and nurses in Sweden which showed: 1. there is unclear decision-making and understanding of a patient’s situation, 2. lack of accepting the failure of therapy by the treating team 3. poor concept and understanding about palliative care, and 4. keeping the patient in a state of uncertainty which causes more suffering than necessary.[1]

The costs of cancer treatment have increased due to advancements in new drugs and equipments, whereas these do not necessarily improve the cure or survival in advanced stages. Hence, there have been recent evaluations to reduce healthcare costs.[4] Palliative care is aimed to relieve symptom burden with limited economic impact. Specialized palliative care has the potential to reduce costs for the patient and family.[12] Palliative care is associated with a significant reduction in costs and can generate substantial savings to the health system by cost avoidance in terms of reduced hospitalization and interventions.[13] Compared to anticancer therapy, the cost burden can be limited by implementing programs for transferring palliative care technology to home settings.[14]

## THE PLACE OF PALLIATIVE CARE

Significant medical progress to achieve cure and improve survival has been made in the last 20 years for most chronic diseases (cancer, HIV/AIDS, neuropsychiatry disorders, age-related diseases). However, it is realized that the pursuit for advancement in medical technology and treatment modalities do have their limitations. This is best exemplified in cancer care. *“Some cancers are almost always curable, some are usually incurable, and most cancers fall in between.”.*[15]

By current global estimates, nearly 7 million out of the 11 million newly diagnosed cancer patients present in advanced stages.[16] More important to our cancer physicians, is the knowledge that nearly 50% of all new cancer cases will die within 12 months of diagnosis, and the five-year cure rates for all cancers in the developing and developed countries range from 30–60%.[17] The lower cure rates are seen in most low- and middle-income countries of the world. By the year 2020, up to 70% of the 20 million new cancer patients will be detected in these developing countries and a similar proportion i.e.70% of all cancer deaths will occur in these regions of the world.[18] It is our onus, while working in high-technology and cost-intensive specialties like radiation oncology and medical oncology, that we learn the medical policies of *‘utility versus futility’*. This will avoid futile cancer-directed therapy in our daily practice. Studies at the end of life in advanced cancer patients have shown that a patient-physician discussion to initiate palliative care and focus on improving a patient’s quality of life is likely to reduce the healthcare system costs. For example, estimates showed that this can be reduced by $76 billion in USA.[19] The proportion of cancer patients who present in an advanced incurable stage and those who do not show response during or after a course of CDT should be facilitated for the transition from an objective of achieving cure to receiving cost-effective palliation. This will reduce unnecessary medical care costs in a country like India where the majority of patients pay out of their pocket.

## PATIENT-PHYSICIAN INTERFACE IN ACHIEVING TRANSITION

How do we prepare the healthcare system and our professionals to decide and plan for a shift in their objectives from curative management to palliative care? It is realized that our medical curriculum and training are geared to impart all information and skills towards the goals of cure and disease control.[3,4,15,19] Any admission of ineffective therapy by the doctor is considered as admitting defeat. Yet, in clinical practice, physicians and nurses will encounter a case scenario similar to Mr. KS in their daily professional responsibility. *A disease like cancer will be detected in different forms and contemporary CDT can often be futile.*

The treatment phases of a patient will depend upon disease condition/stage, patient’s general health, and preference of the patient and family. When the disease is far advanced, is not responding to the cancer treatment modalities, and the patient’s general health is low, aggressive therapy is not going to benefit and may lead to unnecessary toxicities. The value of evaluating the treatments in cancer has shown that there will be a situation where CDT can become inappropriate over treatment if they result only in disease-related and iatrogenic harm to the patient.[2,20] The majority of these incurable cancer patients will live for a reasonable length of time ranging from a few weeks to more than six months. For these patients, quality cancer care includes access to palliative care and increasing evidence suggests that timely enrollment can increase quality of life for patients and their caregivers in the family.[2,3] It is important that medical attention to focus on symptom control and psychosocial issues is initiated at an appropriate time. If this decision is made too late, meaningless treatment can result in suffering for the patient.[3]

Cancer care model has developed *Palliative Care* as a medical specialty, which does not aim for disease control, and will address the patient’s physical and psychosocial symptoms and improve the quality of life.[21] It is also appreciated that palliative care can be delivered concurrently with anti-cancer treatments, when there is scope to reduce the tumor burden and relieve distressing symptoms.[2] Medical professionals should communicate this information and lay emphasis on securing effective palliation in relieving difficult symptoms, and helping patients maintain their dignity. Doctors should clearly convey to patients and families that inappropriate prolongation of treatment is undesirable.[1,20] When a physician is of the opinion that cure is no longer an option, the decision of a patient to refuse further oncological treatment seems easier.[1,22] The transition to palliative care becomes smooth when the doctors communicate the disease status and prognosis, the questions and emotions of patients and families are adequately answered, and a discussion about palliative care is done.[23] For an improved patient-physician relationship, the values and wishes of a terminal-stage patient (and relatives) should be adequately evaluated by the medical team.[22]

## OVERCOMING THE BARRIERS IN IMPLEMENTING A TRANSITION

Yet, the shift in cancer care trajectory is not simple. The cancer patient does not always go through a conventional model of curative, palliative, and terminal phases of medical care. The boundaries are unclear in a clinical setting. It is difficult to find clear guidelines and it is noted that often there are no national standards for admission to palliative care.[1] Hence, considerable delay occurs in referring a patient for palliative and end-of-life care.[18,20,21,24]

There are several barriers to the medical community’s effective implementation of a transition from curative to palliative care

- physician’s inability to evaluate the futility of aggressive therapy
- patient or relative does not want to stop anti-cancer treatment
- a reluctance to communicate the real situation of incurable stage
- unavailability of palliative care facility and/or right drugs
- Cultural, linguistic and religious differences

When the treating doctors and nurses face a patient with advanced incurable disease, it is a sensitive decision-making process to forego anti-cancer therapy and to share the right information with patients and caregivers in the family regarding the benefits of palliative care vis-à-vis the harm of aggressive treatment.[3,23,25] Important issues in the transition from curative treatment to palliative care are agreement, timing, and decision-making.[3] The availability of health professionals trained in the delivery of palliative care is a major limitation in many countries. Compared to disease-oriented focus in cancer treatments, the patient-centered approach is essential to palliative care.[25,26] Effective palliative and supportive care is considered as much a right of incurable and symptomatic patients and as well an obligatory component of health service delivery. Millions of patients with terminal cancer often suffer through unrelieved pain due to lack of proper referral, drugs, or trained medical personnel.[24]

In order to achieve a suitable and humane continuity of care till the last phase of life, the understanding about transition as a medical judgment of *‘benefit versus burden’* should be improved.[27]

Several measures are necessary:

- cancer specialists and other health personnel dealing with chronic diseases should learn about the basics of palliative care
- improve communication skills and enhance participation of patient/relative in medical decision-making
- integrative model of healthcare system ranging from active treatment modalities to palliative care setup
- identify medical, social, cultural, and geographic hurdles in developing palliative care

In the Indian context, the socio-cultural aspects of a patient and family members become relevant while communicating the news about incurability, estimated prognosis and the shift of a medical goal from CDT to symptom alleviation and palliative care. Therefore, family members can receive the full medical information, whereas the patient receives information gradually and often partially, depending on their preferences.[28]

## SUMMARY

The crisis of living with an incurable illness can be lessened for patients (and the family members) with greater understanding of the needs for change in treatment goals and care.[3,4,19,20] Communication about prognosis and transition in the goals of medical care should be a part of the overall oncologic care plan.[2] For patients who fail to respond to the primary CDT, second-line treatments, in the form of chemotherapy, radiotherapy and surgery, have been explored with limited benefits. It is often seen that medical interventions in the face of a progressive and unresponsive disease add to the costs of manpower, infrastructure and budgetary provisions for any country. Doctors have an onus to reduce the burdens of financial expenses on the patients and healthcare system. The medical community should endeavor to implement resource-level-appropriate palliative care programs within the existing health systems.[4,17,18] In the developing countries of Asia and Africa, there is a need to identify the patients who will require the transition from curative to palliative care approaches. The benefits of palliative and supportive care for a cancer patient have shown good scientific foundations, and such approaches can be implemented for other terminal patients of HIV, neurology, cardiology, and geriatrics. This will avoid unnecessary healthcare costs and distress for the patient and family.

## References

[1] Lofmark R, Nilstun T, Bolmsjo IA (2007). From cure to palliation: concept, decision and acceptance. J Med Ethics.

[2] Finlay E, Casarett D (2009). Making difficult discussions easier: Using prognosis to facilitate transitions to hospice. CA Cancer J Clin.

[3] Bolmsjo IA, Nilstun T, Lofmark R (2007). From cure to palliation: Agreement, timing and decision making within the staff. Am J Hospice Palliat Med.

[4] Schickedanz A (2010). Of value: A discussion of cost, communication and evidence to improve cancer care. Oncologist.

[5] Stinchcombe TE, Socinski MA (2008). Consideration for second line therapy of non-small cell lung cancer. Oncologist.

[6] Neuberger MA, Hoverman JR, Kolodziej M, Reisman L, Gruschkus SK, Hoang S (2010). Cost effectiveness of evidence-based treatment guidelines for the treatment of non–small-cell lung cancer in the community setting. J Oncol Pract.

[7] Hassett MJ, O’Malley AJ, Pakes JR, Newhouse JP, Earle CC (2006). Frequency and cost of chemotherapy related serious adverse effects in a population sample of women with breast cancer. JNCI.

[8] Javle M, Hsueh CT (2010). Recent advances in gastrointestinal oncology-updates and insights from the 2009 annual meeting of the American society of clinical oncology. J Hematol Oncol.

[9] Lutz ST, Chow EL, Hartsell WF, Konski AA (2007). A review of hypofractionated palliative radiotherapy. Cancer.

[10] Joseph K, Tai P, Wu J, Barnes E, Levin W (2010). Workshop report: A practical approach and general principles of re-irradiation for in-field cancer recurrence. Clin Oncol.

[11] Bauman JE, Mulligan MS, Martins RG, Kurland BF, Eaton KD, Wood DE (2008). Salvage lung resection after definitive radiation(>59 Gy) for non-small cell lung cancer: Surgical and oncologic outcome. Ann Thorac Surg.

[12] Boni-Saenz AA, Dranove D, Emanuel LL, Lo Sasso AT (2005). Price of palliative care: Towards complete accounting of costs and benefits. Clin Geriatr Med.

[13] Smith TH, Cassel JB (2009). Cost and non-clinical outcomes of palliative care. J Pain Symptom Manage.

[14] Witteveen PO, van Groenestijn MA, Blijham GH, Schrijver AJ (1999). Use of resources and costs of palliative care with parenteral fluids and analgesics in the home setting for patients with end stage cancer. Ann Oncol.

[15] Larsson S (2003). A career in oncology. Br Med J.

[16] Globocan 2002. Cancer incidence, mortality and prevalence.

[17] (2002). National Cancer Control Programmes: Policies and managerial guidelines.

[18] Huerta E, Grey N (2007). Cancer control opportunities in low-and middle-income countries. CA Cancer J Clin.

[19] Zhang B, Wright AA, Huskamp HA, Nilsson ME, Maciejewski ML, Earle CC (2009). Health care costs in the last week of life: Associations with end-of-life conversations. Arch Intern Med.

[20] Braun UK, Beyth RJ, Ford ME, McCullough LB (2007). Defining limits in care of terminally ill patients. Br Med J.

[21] (1990). World Health Organization. Cancer pain relief and palliative care.

[22] van Kleffens T, van Leeuwen E (2005). Physician’s evaluations of patients’ decisions to refuse oncological treatment. J Med Ethics.

[23] Goelz T, Wuensch A, Stubenrauch S, Bertz H (2010). Addressing the transition from curative to palliative care: Concept and acceptance of a specific communication skills training for physicians in oncology-COM-ON-p. Onkologie.

[24] Brennan F, Carr DB, Cousins M (2007). Pain management: A fundamental human right. Aesth Analg.

[25] Grant M, Elk R, Ferrel B, Morrison RS (2009). Current status of palliative care-clinical implementation, education and research. CA Cancer J Clin.

[26] Saraiya B, Bodnar-Deren S, Leventhal E, Leventhal H (2008). End of life planning and its relevance for patients’ and oncologists’ decisions in choosing cancer therapy. Cancer.

[27] Cherney N, Catane R, Kosmidis P (2003). Editorial. ESMO takes a stand on supportive and palliative care. Ann Oncol.

[28] Morita T, Akechi T, Ikenaga M, Kizawa Y, Kohara H, Mukaiyama T (2004). Communication about the ending of anticancer treatment and transition to palliative care. Ann Oncol.

